# Characteristics and Risk of Adverse Mental Health Events Amongst Users of the National Overdose Response Service (NORS) Telephone Hotline

**DOI:** 10.1007/s11469-024-01285-1

**Published:** 2024-03-25

**Authors:** Dylan Viste, Will Rioux, Nathan Rider, Taylor Orr, Nora Cristall, Dallas Seitz, S. Monty Ghosh

**Affiliations:** 1 Department of Medicine, Cumming School of Medicine, University of Calgary, 205 3500 Varsity Drive NW, Calgary, AB T2L 1Y3 Canada; 2 Department of Medicine, Faculty of Medicine & Dentistry, University of Alberta, Edmonton, AB Canada; 3 Public Health Surveillance and Informatics, Alberta Health Services, Calgary, AB Canada; 4 Department of Internal Medicine, Faculty of Medicine & Dentistry, University of Alberta, Edmonton, AB Canada

**Keywords:** Virtual overdose response, Opioid Epidemic, Overdose Reversal, Harm Reduction, Concurrent substance use and mental health disorders, Methamphetamine psychosis management, Verbal De-escalation psychosis, Peer support

## Abstract

The National Overdose Response Service (NORS) is a Canadian mobile or virtual overdose response hotline intended to prevent drug overdose deaths but has unexpectedly received mental health related calls, including adverse mental health events. Our study aimed to examine these occurrences and caller characteristics predictive of adverse mental health outcomes. Using the NORS call dataset, we conducted a descriptive representation of mental health occurrences and mental health emergencies along with correlative statistics. We found that NORS had received 2518 mental health calls, with 28 (1.1%) being adverse events. Men, rural callers, polyroute substance consumption and history of overdosing were found to have increased odds of having an adverse mental health event, while being from Quebec, using non-standard consumption routes and using the line between 50 and 99 times were found to decrease odds. This supports the utility of overdose prevention hotlines to also support people experiencing adverse mental health situations and reduce harm for individuals with mental health and/or substance use disorders.

Since 2016, overdose rates have almost tripled in Canada, rising from a rate of 6.8 to 20.3 deaths per 100,000 people at its peak in 2021 (Health Agency of Canada, 2022). People who use drugs (PWUD) face a more toxic drug supply in recent years (Hu et al., 2022) and high rates of co-occurring mental disorders, including depression and anxiety (Genberg et al., 2019; Rogers et al., 2021; Santo et al., 2022). Consequently, there are increasing calls for integrated support for combined mental health and addiction support with novel strategies to help address the disproportionate harms seen within this population (Yule & Kelly, 2019).

Because the majority of North American overdose fatalities occur while using alone (Belzak & Halverson, 2018; O’Donnell et al., 2021), Canada’s National Overdose Response Service (NORS) was established in 2021 (National Overdose Response Service, 2021). NORS is a mobile overdose response service hotline or virtual supervised consumption site that allows people from across Canada to connect via phone to a peer operator with lived experience of substance use. NORS is one of several similar overdose detection and response technologies that have recently become available (Loverock et al., 2023). This peer operator collects key information, including a client’s physical address, and then virtually “spots” the caller while they use their substance of choice. During these calls, operators often maintain friendly conversation while the client uses substances. If at any point the client becomes unresponsive during their call, the peer operator will alert emergency medical services (EMS) or a predetermined contact person (Matskiv et al., 2022; Perri et al., 2021; Rioux et al., 2023). Since the start of the program in December 2020 to April 2023, NORS has serviced 6528 calls and has handled 77 overdose/drug poisoning emergencies with no fatalities (Viste et al., 2023).

Although not an original objective of NORS, the program has increasingly focused on concurrently managing mental health-related calls (Viste et al., 2023). NORS receives these calls even though there are other free provincial and national mental health and distress hotlines available, including those dedicated to suicide prevention as well as resource provision (Crawford, 2021). Mental health calls have line operators providing peer support to the callers independently or while performing virtual “spotting” (Mercer et al., 2021). Callers often phone NORS to discuss their lives and past traumas they have experienced or seek emotional support (Rider et al., 2023). While current evidence remains limited, peer support services within addiction realms have demonstrated benefits including substance use, treatment engagement, the transmission of sexually transmitted and blood-borne illness and secondary behaviours including self-efficacy and craving control (Tracy & Wallace, 2016). Similarly mental health peer support has been demonstrated to reduce healthcare usage alongside additional usage benefits (Repper & Carter, 2011). The hotline format of NORS facilitates supportive conversations between operators and callers while they use substances (Ritchie & Ghosh, 2022). Other mental health supports that NORS delivers include “mental health first aid” when the caller is in distress and requires emotional support, debriefing, or guided relaxation techniques to manage their anxiety or agitation (Rider et al., 2023). Other mental health calls have pertained to suicidal ideation or thoughts of harm to others, resulting in NORS operators following processes for appropriate response, such as EMS dispatch or referral to crisis lines (Viste et al., 2023). Additional mental health adverse events managed by NORS include methamphetamine-induced psychosis and agitation through means of verbal de-escalation (Rider et al., 2023).

Our study aimed to determine the characteristics of callers receiving mental health support from the NORS line and determine which of these characteristics are associated with having an adverse mental health event. The results of this study will inform strategies to support the mental health needs of NORS clients and provide useful information for the planning, implementation, and quality improvement of NORS and similar services.

## Methods

A retrospective mixed methods analysis was conducted using routinely collected NORS administrative call log data collected between December 15, 2020, and April 30, 2023.

### Dataset

The raw data was recorded electronically in a call log primarily used by NORS for reporting to various stakeholders and funders, as well as for internal quality improvement and research purposes. NORS collects this information in a manner that preserves privacy and protection of personal health information. Consequently, the data does not include individual identifiers such as legal names or phone numbers. An anonymized unique caller code identifier is used instead which facilitates the tracking of repeat callers, including their key demographics including gender, age, and some limited geographic information. During a call, operators note the caller’s full physical address as a transient record for emergency response purposes only; however, only the city name is permanently stored. Operators also record the type and route of illicit substance used, the amount of substance used, and the type of service utilized (supervised consumption, peer support, or resource referral). Beginning in July 2023, collection of other demographic data (e.g. gender identity and Indigenous identity) began voluntarily.

### Call Inclusion Criteria

Only client calls that were for mental health reasons were extracted and included in this analysis. Mental health calls were defined as calls that:

1. Were listed in the call log database as containing any aspect of peer support, mental health support, or mental health first aid
2. Indicated an adverse mental health component (e.g. suicidal ideation, psychosis)
3. Described a mental health or peer support activity (i.e. verbal comforting) within the free text operator notes

Because NORS did not initially expect to provide mental health support, many calls (especially early ones) that contained mental health activities were not categorized as mental health calls by the operators due to data field limitations. Thus, the mental health nature of these calls was assessed using the free text operator notes. One researcher (DV) reviewed the operator notes to find any missing uncategorized mental health calls.

### Call Risk Indexing

To appropriately understand the level of risk of mental health calls, three reviewers (DV, NS, and WR) categorized all mental health calls into low, medium, and adverse events, based on the degree of perceived danger (to the caller themselves or others). A physician with subspecialty training in addictions (MG) then reviewed the rankings to ensure their validity and inter-rater reliability. These categories were classified as follows:

(i) Low risk—routine peer support or mental health calls with no adverse events; operator notes did not indicate any immediate safety concerns.
(ii) Medium risk—calls dealing with distress or non-life threatening situations, usually requiring the operators to verbally de-escalate the situation with no imminent risks identified by the end of the call.
(iii) Adverse mental health events—calls where the caller was at imminent risk of harm to themselves or others due to mental health concerns (e.g. suicidal ideation and psychosis).

### Adverse Events

Calls identified as mental health adverse events required one of the following: (1) emergency medical services (EMS) dispatch, (2) dispatch of the client’s designated contact, or (3) verbal de-escalation of a situation presenting an imminent risk of harm managed by a NORS line operator. Designated contacts are lay responders who act as an alternative to formal EMS for those clients who prefer not to have professional services attend to them in the event of the need to contact someone for their safety. These were automatically categorized as adverse events regardless of the presence of corroborative operator notes.

Calls that ended up being transferred to another mental health distress hotline and not emergency dispatch were included as medium-risk calls since it was sometimes difficult to assess the level of severity due to lack of information.

### Mental Health Categories

To better understand the interactions and types of mental health calls NORS encountered, three coders (DV, NS, and WR) performed thematic content analyses using an inductive open coding scheme to identify recurring mental health topics of conversation or activities associated with each call. Codes were determined by the following: (1) categorized mental health-related call topics from a select-all-that-apply menu built into the operator call log (i.e. “peer support”, “mental health first aid”, and “resources provided”) and (2) the free text responses in the operator notes for topics that did not appear in the menu. Each reviewer generated their codes, and afterwards, similar groups were joined together to make the final categories.

### Statistical Analysis

To further explore potential personal and call risk factors for adverse mental health events, we conducted a multivariate mixed-effects logistic model analysis (Li et al., 2011). A mixed-effects logistic model was chosen to control for a lack of independence between call logs because multiple call logs could be from the same caller (Li et al., 2011)(UCLA: Statistical Consulting Group, 2021). The model’s random effect variable was grouped by each call record's unique caller ID. Call records that could not be determined as unique individuals were removed (*n* = 57, 2.2% of total data).

To address missing data within our demographic indicators (age, gender, town size, and region), multiple imputation by chained equations (MICE) was used (Azur et al., 2011). In total, 165 records (6.5%) were missing their age, 193 records (7.6%) were missing their gender identity, 176 records (6.9%) were missing their town size, and 53 records (2.1%) were missing their region. The dataset was imputed 30 times over 20 iterations. The multivariate logistic mixed-effects model was run on all 30 imputed datasets, and their model coefficients were then averaged to create a final model. Odds ratios and 95% confidence intervals predicting the likelihood of an adverse mental health outcome were calculated using the final model’s averaged coefficients and standard errors. The model’s reference groups were chosen based on the characteristics with the highest call frequencies based on the total number of calls in the entire data set (Viste et al., 2023). Modelling was performed using R version 4.3.0 with the packages “mice” and “lme4” (Bates et al., 2023; van Buuren & Groothuis-Oudshoorn, 2011). The forest plot for Fig. 4 was created using Tableau 2023.3.

## Results

### Mental Health Calls

**Fig. 1:**
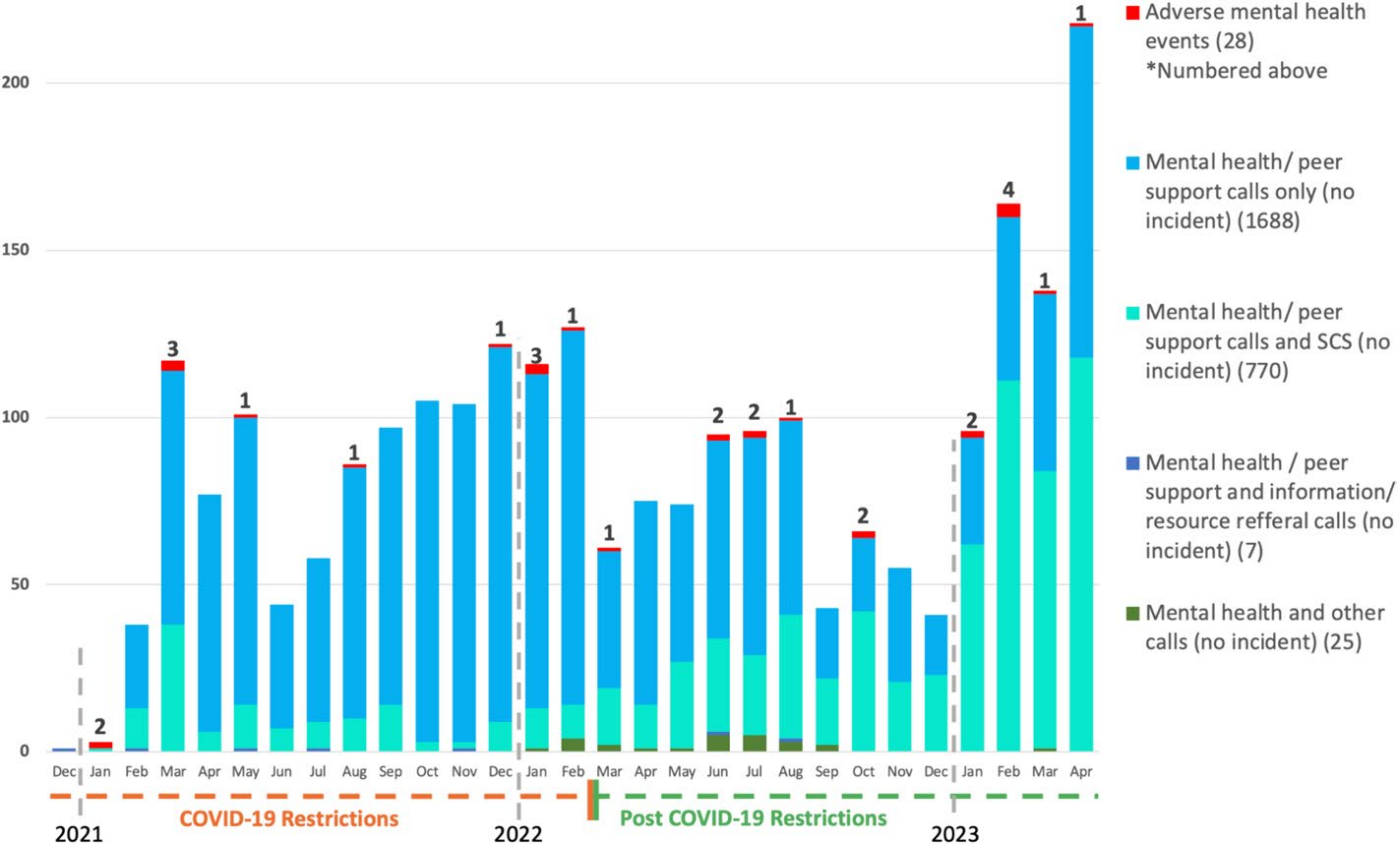
Monthly mental health calls received by NORS; December 2020–April 2023 (*N* = 2518)

Between December 15, 2020, and April 30, 2023, NORS received 6528 calls, of which 2518 (38.6%) were identified as having a mental health component. Figure 1 shows the uptake of mental health services at NORS over time. In general, the number of overall calls has increased over time with a slight lull during the latter part of the COVID-19 pandemic. Calls for mental health reasons appear to represent an increasing proportion of total call especially towards the end of 2023. Notably, there were no differences between mental health calls pre-pandemic as well as after-pandemic restrictions were lifted.

**Fig. 2:**
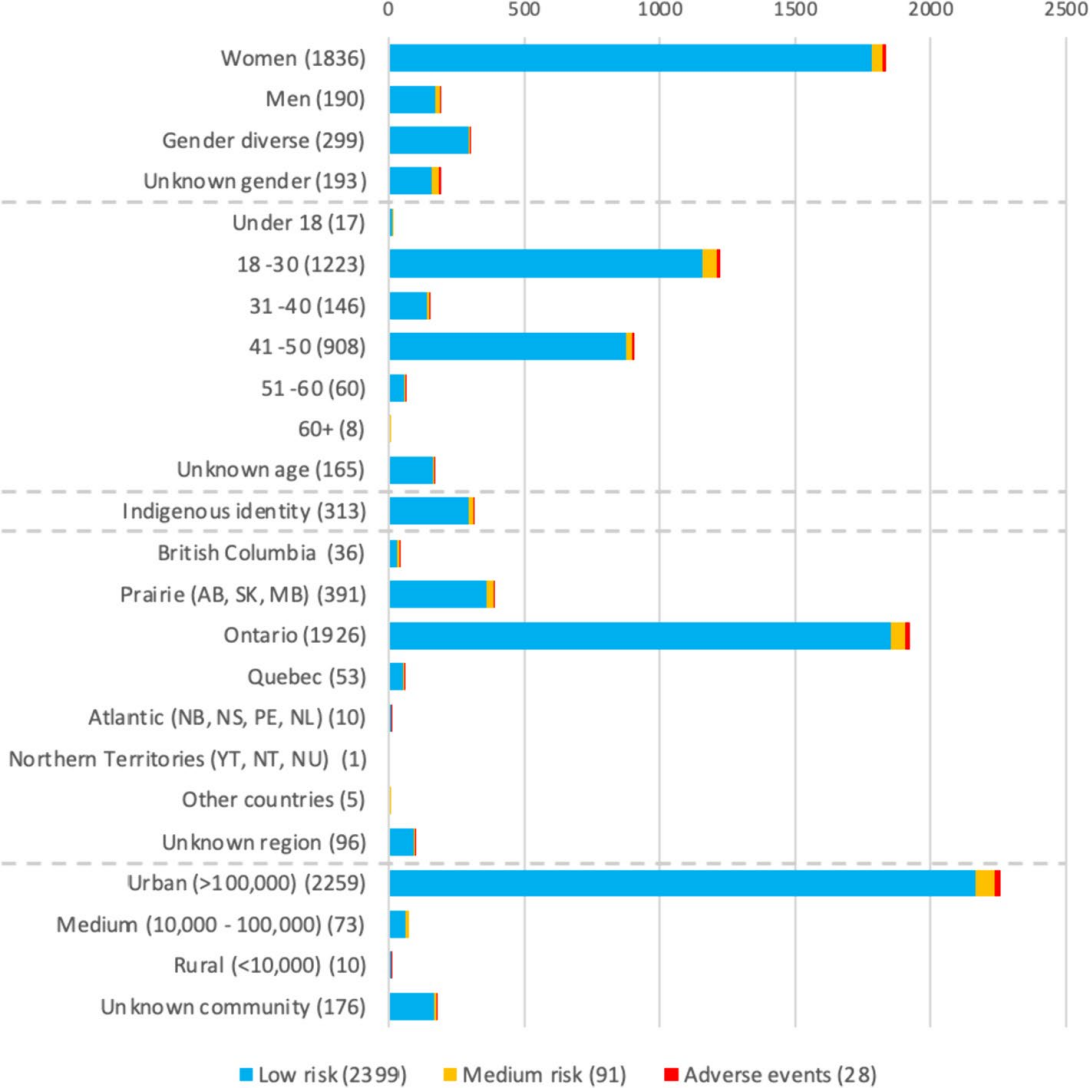
NORS caller demographic characteristics for mental health calls; December 2020–April 2023 (*N* = 2518)

**Fig. 3:**
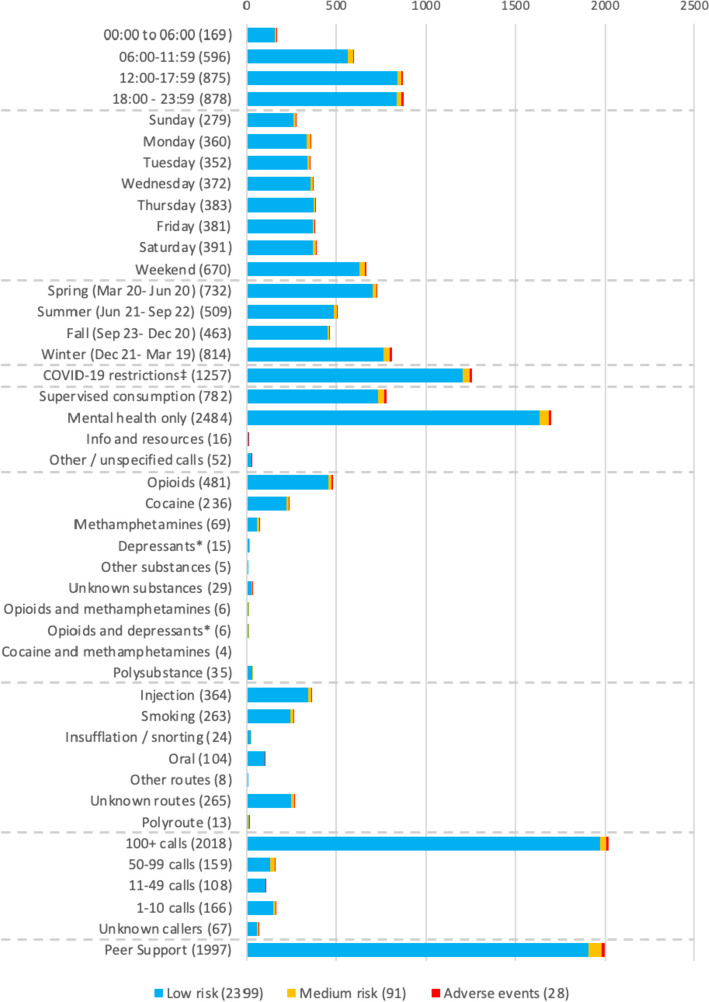
NORS call characteristics for mental health calls; December 2020–April 2023 (*N* = 2518). ^**‡**^The duration of the Canadian COVID-19 restrictions was defined as between March 1st, 2020, and March 1st, 2022. ^*^Depressants include depressants such as benzodiazepines and alcohol

Mental health calls were overwhelmingly low risk (2399, 95.3%), but 91 (3.6%) were considered medium risk, and 28 (1.1%) were considered as clear adverse mental health events (Figs. 2 and 3) requiring escalating support. Of the 2518 mental health calls, 1836 (72.9%) of calls came from individuals identifying themselves as women, 1223 (48.5%) from people aged 18–30, 1926 (76.4%) from the province of Ontario, and 2259 (89.7%) from large urban communities in Canada, and there was relative even distribution across days of the week and season and 844 calls occurring during the afternoon 1200–1800 h (34.7%) (Table 1).

**Table 1:** Risk categories of calls by caller and call characteristics

Characteristic	Low-risk	Medium risk	Adverse events	% of total calls	% of total adverse events
**Total (2518)**	**2399**	**91**	**28**	**100%**	**1.1%**
*Gender*					
Women	1782	42	12	72.9%	42.9%
Men	168	19	3	7.5%	10.7%
Gender diverse	292	4	3	11.9%	10.7%
Unknown gender	157	26	10	7.7%	35.7%
*Age*					
Under 18	16	1	0	0.7%	0.0%
18–30	1158	50	15	48.6%	53.6%
31–40	139	6	1	5.8%	3.6%
41–50	876	23	9	36.1%	32.1%
51–60	55	4	1	2.4%	3.6%
60 +	6	2	0	0.3%	0.0%
Unknown age	158	5	2	6.6%	7.1%
Indigenous identity	295	17	1	12.4%	3.6%
*Province*					
British Columbia	33	2	1	1.4%	3.6%
Prairie (AB, SK, MB)	358	30	3	15.5%	10.7%
Ontario	1854	52	20	76.5%	71.4%
Quebec	51	1	1	2.1%	3.6%
Atlantic (NB, NS, PE, NL)	8	1	1	0.4%	3.6%
Northern territories (YT, NT, NU)	1	0	0	0.0%	0.0%
Other countries	4	1	0	0.2%	0.0%
Unknown region	90	4	2	3.8%	7.1%
*Community type and size:*					
Urban (> 100,000)	2166	70	23	89.7%	82.1%
Medium (10,000–100,000)	59	14	0	2.9%	0.0%
Rural (< 10,000)	8	0	2	0.4%	7.1%
Unknown community	166	7	3	7.0%	10.7%
*Time of day of call:*					
00:00 to 06:00	154	12	3	6.7%	10.7%
06:00 to 11:59	564	29	3	23.7%	10.7%
12:00 to 17:59	844	21	10	34.7%	35.7%
18:00 to 23:59	837	29	12	34.9%	42.9%
*Day of the week:*					
Sunday	258	15	6	11.1%	21.4%
Monday	332	22	6	14.3%	21.4%
Tuesday	339	12	1	14.0%	3.6%
Wednesday	356	12	4	14.8%	14.3%
Thursday	374	6	3	15.2%	10.7%
Friday	370	8	3	15.1%	10.7%
Saturday	370	16	5	15.5%	17.9%
Weekend	628	31	11	26.6%	39.3%
*Season:*					
Spring	702	24	6	29.1%	21.4%
Summer	484	19	6	20.2%	21.4%
Fall	450	10	3	18.4%	10.7%
Winter	763	38	13	32.3%	46.4%
COVID-19 restrictions^‡^	1203	41	13	49.9%	46.4%
*Type of call:*					
Supervised consumption	734	36	12	31.1%	42.9%
Mental health only	1637	51	14	67.6%	50.0%
Info and resources	5	2	1	0.3%	3.6%
Other/unspecified calls	23	2	1	1.0%	3.6%
*Type of substance used:*					
Opioids	456	17	8	19.1%	28.6%
Cocaine	221	13	2	9.4%	7.1%
Methamphetamines	56	10	3	2.7%	10.7%
Depressants^*^	13	2	0	0.6%	0.0%
Other substances	5	0	0	0.2%	0.0%
Unknown substances	25	3	1	1.2%	3.6%
Opioids and methamphetamines	5	1	0	0.2%	0.0%
Opioids and depressants^*^	5	1	0	0.2%	0.0%
Cocaine and methamphetamines	4	0	0	0.2%	0.0%
Polysubstance	31	4	0	1.4%	0.0%
*Route of substance used:*					
Injection	341	18	5	14.5%	17.9%
Smoking	244	15	4	10.4%	14.3%
Insufflation/snorting	22	2	0	1.0%	0.0%
Oral	99	1	4	4.1%	14.3%
Other routes	8	0	0	0.3%	0.0%
Unknown routes	245	17	3	10.5%	10.7%
Polyroute	8	4	1	0.5%	3.6%
*Frequency of calls:*					
100 + calls	1970	37	11	80.1%	39.3%
50–99 calls	129	27	3	6.3%	10.7%
11–49 calls	101	4	3	4.3%	10.7%
1–10 calls	145	15	6	6.6%	21.4%
Unknown callers	54	8	5	2.7%	17.9%
Peer support/active listening provided	1907	74	16	79.3%	57.1%
No peer support provided	492	17	12	20.7%	42.9%

^**‡**^The duration of the Canadian COVID-19 restrictions was defined as between March 1st, 2020, and March 1st, 2022

^*^Depressants include depressants such as benzodiazepines and alcohol

Cumulatively, callers used overdose monitoring and mental health support together 782 (31.0%) times; with the most common substance used being opioids (481, 19.1%) and the most common route of consumption being injection (364, 14.4%). The vast majority of mental health calls were individuals seeking peer support (1997, 79.3%), followed by mental health supports including substance related psychosis de-escalation, verbal management of anxiety or managing schizophrenia (21, 0.83%), and lastly discussing suicide or self-harm (22, 0.87%). Another feature of note was that (404, 16.0%) of calls were explicitly not using any substances and thus solely calling for mental health support. Additionally, fifteen (0.60%) were calling for support/ distraction while experiencing substance-related withdrawal. Sixty (2.38%) of calls noted that the client had expressed appreciation for having a positive therapeutic relationship with NORS line operators (for example, requesting a specific operator). Lastly, some calls (38; 1.51%) required boundary setting (usually due to excessive call volume from a single client or secondary to sexually inappropriate conversation topics).

### Adverse Events

In total, 28 adverse mental health events occurred. Of these, ten (35.7%) required a physical response from either EMS or a designated contact (Table 2). The remaining 18 (64.3%) were managed by the NORS staff verbally. It should be noted that NORS staff stay on the phone providing verbal instruction and support to the caller until help arrives thus staff verbal assistance is normally present even following activation of EMS or a designated contact. Table 2 describes (with examples) what types of in call adverse events encountered. Table 3 shows the number of occurrences for the various outcomes, responses, and situations encountered during these adverse event calls. First responders were dispatched eight times (28.6%), while four callers required verbal assistance after EMS refusal (14.3%).

**Table 2:** Details of mental health adverse events on the NORS line from December 2020 until April 2023. Details for verbally managed calls are excerpts from line operator notes

NORS: examples of types of mental health adverse events based on hotline operator notes: Dec 2020–Apr 2023
Verbally managed
Verbally assisting people who refuse EMS
*Caller was in duress [distress], [had] a panic attack due to crack use which he thought was an OD. Operator offered to call EMS and caller was adamantly not in agreement. Operator got client’s information (address, name, medications, *etc*.) and then talked caller through until they felt safe again*
Call lead to a hospital visit
*Partner called on behalf of girlfriend, suspected suicide attempt. Caller was prompted to take their partner to emerg[ency] and did so*
In-call events
Suicide/self-harm prevention
*Caller suicidal and [making] threats of starting a fire. EMS dispatched*
*History of self-harm. Cut herself too deep, wanted to stay on the phone until help arrived*
Stimulant psychosis
*Unspecified stimulant. Anxiety de-escalation. Conference call with telehealth—recommended immediate EMS. Refused. Referred to family doctor and [suggested] continual use of Lifeguard app*
Extreme anxiety
*Caller had panic attack—going to hospital*
*Had anxiety attack after smoking fentanyl, he woke up his roommate, stayed on the phone with him while he was enroute to the hospital*
Other (domestic violence and human trafficking)
*In between uses, client stated her ex-partner came to the door and started trying to break it down with a knife. Stayed on call in case police intervention was required (with consent). Emotional support*
*They are being human trafficked [and] won’t share location*

**Table 3:** Occurrences of mental health adverse events on the NORS line from December 2020 until April 2023

NORS: details of mental health adverse events: Dec 2020–Apr 2023	
**Outcomes and details**	***N* (%)**
**Total mental health adverse events**	**28 (100)**
Physical responses	10 (35.7)
Total EMS dispatches	8 (28.6)
Total designated contact responses	2 (7.1)
Verbally managed	18 (64.3)
Verbally assisting people who refuse EMS	4 (14.3)
Call lead to a hospital visit	3 (10.7)
In-call events:	
Suicide/self-harm prevention	8 (28.6)
Stimulant psychosis	3 (10.7)
Extreme anxiety	5 (17.9)
Other (domestic violence and human trafficking)	2 (7.1)

### Predictors of Adverse Mental Health Events

**Fig. 4:**
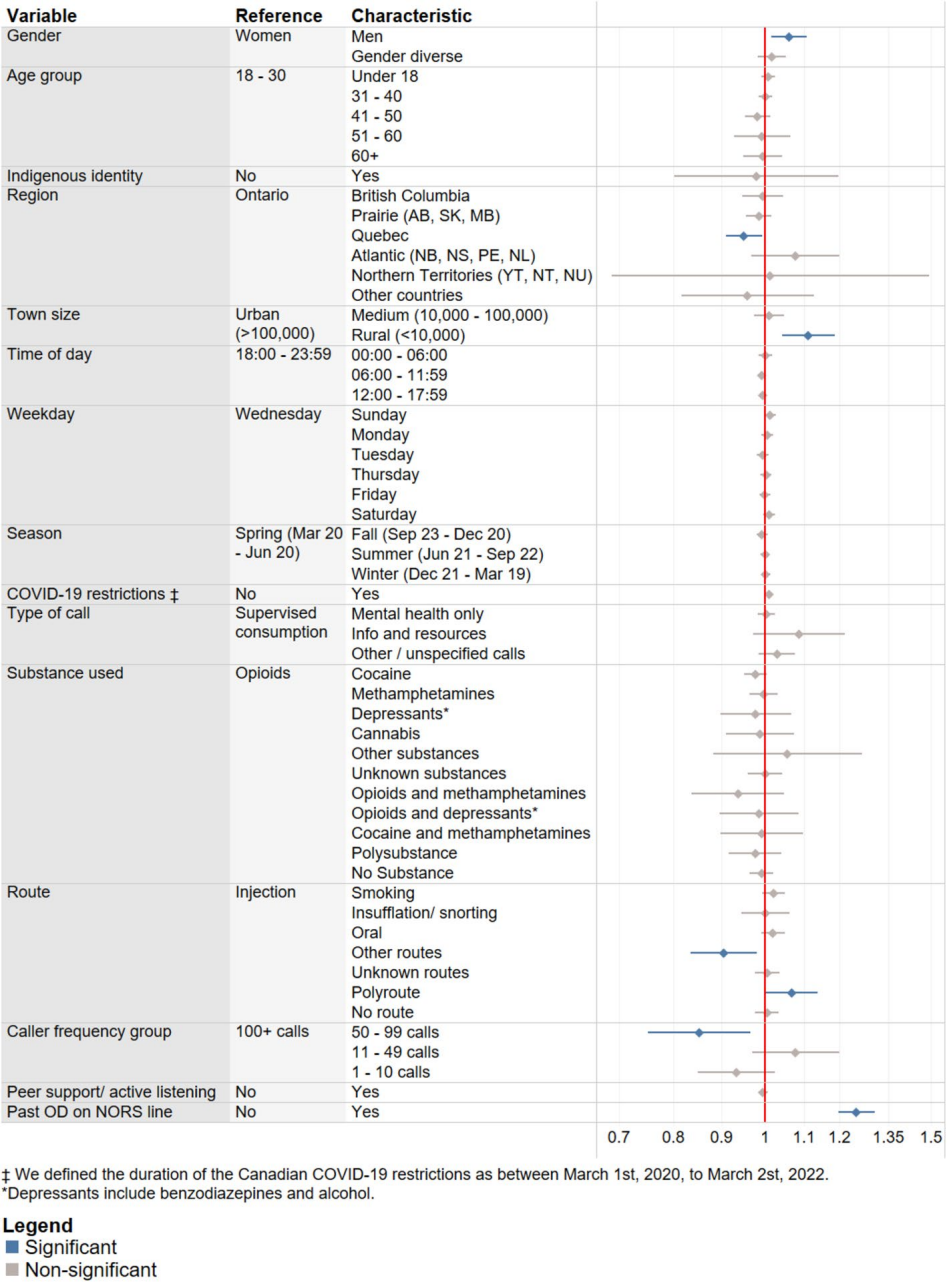
Predictors of adverse mental health events by caller and call characteristics as assessed using the multivariate mixed-effects logistic model

Men had significantly higher odds of experiencing an adverse mental health event than women (OR = 1.06, 95% CI [1.01, 1.11]; Table 3, Fig. 4), while Quebec callers had significantly lower odds than those from Ontario (OR = 0.95, 95% CI [0.91, 0.99]), and callers from rural communities had significantly higher odds compared to urban communities (OR = 1.11, 95% CI [1.04, 1.19]). Compared to callers using injection as their primary route for substance use consumption, callers using a polyroute (multiple routes of consumption) had significantly higher odds of a mental health adverse event (OR = 1.07, 95% CI [1.00, 1.14]), while callers using a route classified as “other” (i.e. rectal, ocular, subcutaneous) had significantly lower odds (OR = 0.90, 95% CI [0.83, 0.98]). Compared to callers who have called the NORS line over 100 times, individuals with a call frequency between 50 and 99 had significantly lower odds of a mental health adverse event (OR = 0.85, 95% CI [0.75, 0.96]); however, callers with fewer than 50 calls did not demonstrate the same trend. Lastly, if the caller had ever experienced an overdose event between December 2020 and April 2023, then their odds of experiencing a mental health adverse event were significantly increased (OR = 1.25, 95% CI [1.19, 1.31]). A complete tabular list of odds ratios is available in Table 4 in the Appendix.

## Discussion

While there are other mental health support lines and phone services in Canada, one unique feature of NORS is that it provides mental health support in addition to harm reduction focused overdose monitoring and response. Substance use and mental health disorders are often concurrent, reflecting overlapping aetiologies (Chang et al., 2023; Forray & Yonkers, 2021). This complexity presents unique challenges to support services such as NORS. Harm reduction programs are often effective points of entry into addiction recovery and mental health support pathways, and the integration of harm reduction services with more traditional medical supports is generally viewed favourably by clients (Bartram, 2021; Chang et al., 2023; Khan et al., 2022). Indeed, given that nearly 39% of calls to NORS have a mental health component (Viste et al., 2023), it would be difficult for NORS to avoid addressing mental health issues entirely.

The beneficial role of peer support in harm reduction is well established. Peers can increase accessibility and improve service equity amongst individuals who would otherwise not engage with health resources through conventional means (Hayashi et al., 2010). Peers in harm reduction settings have demonstrated a unique capacity to manage mental health concerns, especially depression and anxiety amongst substance users within their programs (Hay et al., 2017). Furthermore, peer facilitation enhances referral uptake to various social and health services (Tracy & Wallace, 2016). NORS is peer-run and peer-operated, and the provision of peer support on top of harm reduction is likely a natural extension of their existing monitored consumption services. We further speculate that peer support is a necessary component of a responsive NORS program as many callers may be less likely to access traditional mental health and substance recovery services otherwise.

### *Gender*

Women had a much higher absolute utilization of NORS mental health features, but men had an increased odds of having an adverse mental health event. There is a high baseline utilization of NORS services for supervised consumption amongst women (Viste et al., 2023), and this trend continues for mental health support utilization. Globally, women are more likely to interact with suicide prevention hotlines then men (Krishnamurti et al., 2022). Similarly, women account for a disproportionate amount of mood and anxiety disorders; consequently, they may have a disproportionate need for mental health services on the line (Riecher-Rössler, 2017). Other potential reasons for the high uptake of women and gender diverse callers could be because NORS has 61% women and 27% gender-diverse people as staff (*L. Morris-Miller, personal communication, Jan 10, 2024*) which may help facilitate a welcoming and safe environment and safe. There are additional barriers for women to access physical supervised consumption sites, including concerns with violence and sexual harassment (Fairbairn et al., 2008) which may encourage more use of these lines for substance consumption purposes but with secondary benefits of addressing their mental health concerns and forming relationships. Due to limitations within our sample size and low number of callers identifying as men, there may be a statistical bias towards callers for more emergency related needs. Previous literature has found various factors are associated with delays in psychological help seeking amongst men (Yousaf et al., 2015), which may correlate to greater symptom severity and psychological distress upon presentation to the service line. Future research should examine the differences in use patterns between these demographic groups to elucidate rationales behind the differences in use patterns between both groups.

### Age

While no specific age exhibited significant odds of having an adverse mental health event, most calls came from callers aged 18–30 (48.6%) and 40–60 (36.1%). A study of a large Australian mental health helpline found the odds of being a frequent caller peaked at age 55 (Pirkis et al., 2016), which corresponds to our 40–60 age group, and thus, NORS may just have few unique callers of this age using the line more frequently. It is likely that the large number of calls from people aged 18–30 is explained by this demographic’s higher proportion of ownership, comfort with and regular use of technology (Perrin & Anderson, 2019; Statistics Canada, 2022) meaning NORS is low barrier for them to access.

### Geographic Considerations

Rural callers were found to be significantly more likely to experience a mental health adverse event than people of urban or medium-sized communities. This may be related to the general challenges around social determinants of health (Bright et al., 2022; Cody et al., 2023; Heitkamp & Fox, 2022; Whipple et al., 2023), additional stigma faced by rural substance users (Bright et al., 2022; Cody et al., 2023; Heitkamp & Fox, 2022; Whipple et al., 2023) and because rural callers often have less access to other mental health supports than those in larger communities (Edwards et al., 2023). Furthermore, rural Canadians face additional challenges around transportation, employment opportunities, housing availability, and education access (Henning-Smith, 2020; Kevany & Jones-Bitton, 2020; Webb et al., 2009). One solution, telehealth services, allow for the rapid implementation of some health services in underserved areas (Jong et al., 2019) and evidence supports that these services are being increasingly viewed favourably by rural dwellers, due to reduced burden of travelling and increased effectiveness of technology to deliver care (Xu et al., 2022). These services are being implemented in an attempt to improve access to mental health services in rural areas (Myers, 2019), and the results of our study corroborate those of others which demonstrate that tele-harm reduction services like NORS are facing pressure to provide peer mental health supports and community referrals (Rider et al., 2023). Currently, no mental health support hotline specifically serves rural Canadians, although their rates of suicide attempts are disproportionally higher (Barry et al., 2020). At the time of writing, efforts were being made to consolidate hotline-based mental health support through Canada’s 211 phone line on a provincial and national level.

Provincial variations were present in our findings. The finding that callers from Quebec were less likely to have adverse mental health events compared to those from Ontario is somewhat unexpected. Though this might reflect greater investments in harm reduction policy in Quebec compared to Ontario, the same trend does not appear to be present for British Columbia, which has similar levels of policy investment to Quebec (Hyshka et al., 2017). Since general public opinion in Quebec is also more favourable towards harm reduction compared to the rest of Canada (Wild et al., 2021), Quebec may have different harm reduction service access conditions, which might, in turn, influence the number of callers coming to NORS in distress. Being from Atlantic Canada had nearly significantly greater odds of having an adverse event then from Ontario. This may be secondary to limited access to, or knowledge of, mental health supports in the local area with one recent Canadian mental health report stating 59% of Atlantic Canadians surveyed did not know where or how to find mental health support within their jurisdiction (Canadian Psychological Association, 2021).

### Time, Seasonal Variations, and COVID-19

There was no demonstrable difference in adverse mental health events by time, season, or duration of the COVID-19 pandemic restrictions. While the COVID-19 pandemic brought increased isolation and decreased mental health amongst Canadians due to the lockdown (Pongou et al., 2022), the pandemic also corresponded with the early days of NORS when mental health data was not recorded as effectively. Furthermore, NORS did not overtly offer peer mental health support until the later stages of the pandemic. Due to this complex interplay of factors, it is not possible to draw robust conclusions about the impact of the pandemic on mental health adverse events in the studied population. Seasonal depression is a real phenomenon within Canada, with increased rates of mental health concerns during this period, however the NORS line did not notice any increase during winter months. In regards to phenomena such as the Monday blues (Areni et al., 2011), where there is an increase in dread and anxiety at the start of the work week, we found no changes to mental health utilization between the days of the week.

### Substances and Routes of Consumption

An unexpected outcome was that when compared to consuming opioids, no individual substances were found to have significantly different odds for risk of adverse mental health events. Notably, stimulant-induced psychosis (especially methamphetamine-related stimulant psychosis) is a common and debilitating condition (Grant et al., 2012); however, methamphetamine use was not statistically associated with an increased number of mental health adverse events. Methamphetamine psychosis de-escalation was not an original intention of the NORS line; however, due to the increased number of callers using methamphetamine and concurrent and subsequent psychosis experienced by callers, NORS offers de-escalation assistance, engaging in techniques such as reorientation and reduction of external stimuli (Rider et al., 2023). Compared to injecting substances, other routes of consumption were found to be significantly less likely to be involved in an adverse mental health event. This might be accounted for by the types of substances that are likely to be used in such a manner, though the exact nature of any such relationship is unclear from the available data. The increased likelihood of an adverse mental health event associated with polyroute consumption may be explained by increased overall amounts of substance use during polyroute calls, leading to greater negative effects on caller’s mental state.

### Returning Callers and Behaviours

Clients who called NORS (for any reason) between 50 and 99 times had significantly lower odds of adverse mental health events. We do not have an explanation for this phenomena as no clear trend emerged that would indicate that increased usage would decrease the odds of having an adverse event. The single largest predictor of having an adverse mental health event was having previously experienced an overdose event on the NORS line. The first explanation for this is that some of the adverse mental health events described qualify as both adverse mental health events and overdose events, particularly for purposeful suicide attempts or accidental overdoses that occur when a caller is in extreme mental distress and pushing the limits of their substance consumption. A second explanation is that people who have had an overdose on the NORS line may suffer from a high rate of detrimental social factors such as isolation, heavy daily substance use, housing instability, safety concerns, and financial worries. This could lead them to be both more likely to have an overdose event and to have mental health crises.

### Limitations

A variety of limitations were present in this study. Likely the largest limitation is the quality of the NORS dataset, which suffered from both human error and shifting data entry protocols for mental health data. Additionally, because most callers who suffer adverse mental health events may be quite agitated or distressed from the beginning of the call, data collection may prove difficult. Since an imputation method was used to generate missing data, imputed data might yield greater similarities between groups, resulting in a negative effect on power (Azur et al., 2011). Furthermore, the study design prevented the assessment of potentially averted mental health adverse events and some callers might choose not to disclose mental health information such as suicidal ideation.

### Implications for Mobile Overdose Response Services (MORS)

Much like physical SCSs which support both substance use but provide both peer and clinical support, virtual services like NORS can also provide concurrent support. It should be noted that while the one to one spotting nature of the service may support greater discussion around mental health, this results in a more significant operational and staffing burden than their physical supervised consumption counterparts. Future programs providing similar virtual services should consider providing additional staffing resources to provide peer support around mental health. Additionally, overdose response hotlines and applications will likely require some type of mental health crisis management features, whether through accessible links to crisis lines or operators trained to assist. For overdose response hotlines, creating a safer space where one feels comfortable using illicit substances will likely be done through conversation and connection, and thus mental health concerns are likely to be disclosed. We believe that peer support (and also mental health first aid) will likely be as important as offering overdose monitoring for many MORS. The amount of mental health support being offered by NORS may demonstrate that the broader category of MORS could be a new platform for connecting with people who use substances to traditional mental health or addiction services.

## Appendix

**Table 4:** Odds ratio and 95% confidence interval for the likelihood of a NORS caller experiencing an adverse mental health event based on their characteristics

Characteristic	Odds ratios (95% CI)
**Gender (Ref: women)**	
Men	1.06 (1.01–1.11)*
Gender diverse	1.02 (0.98–1.05)
**Age group (Ref: 18**–**30)**	
Under 18	1.01 (0.99–1.02)
31–40	1 (0.98–1.02)
41–50	0.98 (0.95–1.01)
51–60	0.99 (0.93–1.06)
60 +	0.99 (0.95–1.04)
**Indigenous identity (Ref: non-indigenous)**	
Indigenous	0.98 (0.8–1.2)*
**Region (Ref: Ontario)**	
British Columbia	0.99 (0.95–1.04)
Prairie (AB, SK, MB)	0.98 (0.95–1.01)
Quebec	0.95 (0.91–0.99)*
Atlantic Canada (NB, NS, PE, NL)	1.08 (0.97–1.2)
Northern Territories (YT, NT, NU)	1.01 (0.69–1.49)
Other countries	0.96 (0.81–1.13)
**Community size (Ref: urban (> 100,000))**	
Medium (10,000–100,000)	1.01 (0.97–1.05)
Rural (< 10,000)	1.11 (1.04–1.19)*
**Time of call (Ref: 18:00**–**23:59)**	
00:00–06:00	1 (0.98–1.02)
06:00–11:59	0.99 (0.98–1)
12:00–17:59	0.99 (0.99–1)
**Weekday (Ref: Wednesday)**	
Sunday	1.01 (1–1.03)
Monday	1.01 (0.99–1.02)
Tuesday	0.99 (0.98–1.01)
Thursday	1 (0.99–1.01)
Friday	1 (0.99–1.01)
Saturday	1.01 (1–1.02)
**Season (Ref: spring (Mar 20**–**Jun 20))**	
Summer (Jun 21–Sep 22)	1 (0.99–1.01)
Fall (Sep 23–Dec 20)	0.99 (0.98–1)
Winter (Dec 21–Mar 19)	1 (0.99–1.01)
**COVID-19 restrictions** ^**‡**^ ** (Ref: No.)**	
Yes	1.01 (1–1.02)
**Type of call (Ref: supervised consumption)**	
Mental health only	1 (0.98–1.02)
Info and resources	1.09 (0.97–1.21)
Other/unspecified calls	1.03 (0.98–1.07)
**Substances used (Ref: opioids)**	
Cocaine	0.98 (0.95–1)
Methamphetamines	1 (0.96–1.03)
Depressants†	0.98 (0.9–1.07)
Cannabis	0.99 (0.91–1.07)
Other substances	1.06 (0.88–1.27)
Unknown substances	1 (0.96–1.04)
Opioids and methamphetamines	0.94 (0.84–1.05)
Opioids and depressants^†^	0.98 (0.89–1.08)
Cocaine and methamphetamines	0.99 (0.9–1.1)
Polysubstance	0.98 (0.91–1.04)
No substance	0.99 (0.96–1.02)
**Route (Ref: injection)**	
Smoking	1.02 (0.99–1.05)
Insufflation/snorting	1 (0.94–1.06)
Oral	1.02 (0.99–1.05)
Other routes	0.9 (0.83–0.98)*
Unknown routes	1.01 (0.98–1.04)
Polyroute	1.07 (1–1.14)*
No route	1 (0.98–1.03)
**Caller frequency (Ref: 100 + calls)**	
50–99 calls	0.85 (0.75–0.96)*
11–49 calls	1.08 (0.97–1.2)
1–10 calls	0.93 (0.85–1.02)
**Peer support/active listening (Ref: No.)**	
Yes	0.99 (0.98–1)
**Previous overdose event on NORS line (Ref: No.)**	
Yes	1.25 (1.19–1.31)***

*P*-value: < 0.05 “*”, < 0.01 “**”, and < 0.001 “***”.

^**‡**^For this project, we define the duration of the Canadian COVID-19 restrictions as between March 1st, 2020, to March 1st, 2022.

^**†**^Depressants include depressants such as benzodiazepines and alcohol.

## Declarations

### Data Access

The deidentified data used in this paper is available for research, but not commercial, purposes after obtaining written permission from the National Overdose Response Service (weloveyou@nors.ca ) and the University of Calgary.
